# Effect of cholesterol supplementation on cryosurvival of goat spermatozoa

**DOI:** 10.14202/vetworld.2015.1386-1391

**Published:** 2015-12-12

**Authors:** Sunita Behera, Hiron M. Harshan, K. Lekshmi Bhai, K. N. Aravinda Ghosh

**Affiliations:** 1 Department of Veterinary Gynaecology and Obstetrics, Veterinary College, Bengaluru, Karnataka, India; 2 Department of Animal Reproduction Gynaecology and Obstetrics, College of Veterinary and Animal Sciences, Kerala Veterinary and Animal Sciences University, Pookode, Kerala, India; 3 Department of Animal Reproduction, Gynaecology and Obstetrics, College of Veterinary and Animal Sciences, Kerala Veterinary and Animal Sciences University, Mannuthy, Kerala, India

**Keywords:** buck, cholesterol, cryopreservation, functional membrane integrity, motility, spermatozoa

## Abstract

**Aim:**

Sperm membrane cholesterol influences cryodamage during cryopreservation. The present study was carried out to evaluate the effect of varying cholesterol levels in Tris based extenders on the freezability of sexually healthy Malabari buck semen.

**Materials and Methods:**

A total of 48 ejaculates from two adults healthy sexually healthy Malabari bucks were utilized for the study. The collected and pooled ejaculates were divided into four groups with Group I serving as Control - I, Group II and III were treated with 1 mg and 2 mg of cholesterol-loaded-cyclodextrin (CLC)/120 × 10^6^ spermatozoa, respectively, and Group IV, treated with 1 mg methyl-β-cyclodextrin (MβCD) served as Control - II. Manual freezing was carried out to cryopreserve the treated and control spermatozoa.

**Results:**

Treatment of semen samples with CLC resulted in improved maintenance of sperm motility at pre-freeze and post-thaw stages of cryopreservation without affecting hypo-osmotic swelling response. Treatment of semen with 1 mg of CLC/120 × 10^6^ spermatozoa was observed to be better than treatment with 2 mg of CLC/120 × 10^6^ spermatozoa. In general, MβCD treatment was found to result in significantly lower sperm characteristics than those of Control - I and CLC treatment at pre-feeze and post-thaw stages and when incubated up to 4 h.

**Conclusion:**

Cholesterol treatment of sexually healthy Malabari buck semen was found to hold promise for improving cryopreservability of spermatozoa.

## Introduction

Artificial insemination (AI) has become an inevitable tool in the breeding management of many of the domestic animals. Though, AI has been successfully implemented among the cattle population; it has not been as successful among the goats. Irrespective of many years of research, fertility of frozen semen remains one of the limitations in its acceptability in goat breeding.

Sperm damage occurring during cryopreservation due to cold shock, low-temperature exposure, osmotic stress, oxidative stress, and combinations of these conditions [1]. It has been suggested that the sperm plasma membrane is the primary site of damage induced by cryopreservation and is one of the main reasons for reduced motility and fertility of sperm during cryopreservation [2,3]. Cholesterol is an important component in the regulation of membrane fluidity, aiding in the stabilization of the membrane [4]. Cold shock induces plasma membrane lipid phase transitions, which can be reduced by increasing the proportion of cholesterol within the plasma membrane [5]. In addition, cryopreservation induces cholesterol depletion from plasma membrane, which in turn causes membrane destabilization [6]. Buck spermatozoa are a more prone to cold shock because of lower membrane cholesterol to phospholipid ratio compared to rabbit and humans [7]. In recent times, the concept of cholesterol supplementation as a means of increasing sperm membrane cholesterol content and hence freezability has gained acceptance [8]. The increase in the cryosurvival is attributed to the increased membrane fluidity and broadening of phase transition temperature [9]. Cyclodextrins are cyclic oligosaccharides of glucose, if pre-loaded with cholesterol, can insert cholesterol into cell membranes. Several authors have reported increased cryosurvival rates when boars, bull, buck, ram, and stallion spermatozoa were supplemented with cholesterol.

Hence, a study was carried out to evaluate the effect of varying levels of cholesterol in Tris based extenders on the post-thaw motility and plasma membrane intactness of sexually healthy Malabari buck spermatozoa.

## Materials and Methods

### Ethical approval

The study was conducted after approval of research committee and institutional ethical committee.

### Materials

Cholesterol and methyl-β-cyclodextrin (MβCD) were bioreagent grade and purchased from Sigma-Aldrich, India. All freezing chemicals were of analytical grade purchased from Merck life sciences, India and stains from Himedia, India.

### Preparation of cholesterol-loaded-cyclodextrin (CLC)

CLC was prepared according to the method described by Purdy and Graham [10]. In brief, 500 mg of MβCD was dissolved in 1.0 ml of methanol in a glass test tube. In another test tube, 200 mg cholesterol was dissolved in 1.0 ml of chloroform. An aliquot of 0.225 ml of cholesterol solution was transferred to the MβCD solution and stirred to make a clear solution. The prepared solution was then poured into a glass Petri dish. The solvents were removed under a stream of nitrogen gas. The resulting crystals were kept at room temperature for another 24 h for drying and stored in a glass vial at room temperature until use. The working solution of CLC was prepared by adding 40 mg of CLC to 1.0 ml of tris-citric acid-glucose (TCG) buffer and the solution mixed using a vortex shaker.

### Semen collection and cryopreservation

Semen was collected using artificial vagina from two sexually healthy Malabari bucks (two ejaculates each) maintained at AI center attached to the Department of Animal Reproduction, College of Veterinary and Animal Sciences, Mannuthy, Trissur. Immediately after collection the vials were transferred to a water bath at 37°C in the laboratory and all four ejaculates were pooled after preliminary evaluation.

After preliminary evaluation of semen, the sperm concentration of the 48 ejaculates was determined by hemocytometer. Ejaculate having “++++” mass activity having fast distinct swirl formations (on a scale of 0 to +++++), more than 80% of progressive motile sperm, and density “DDDD” were used for the study. The density of semen sample was graded on a scale of D to DDDD, based on visual examination of opacity of a drop of semen taken on a clean, grease free glass slide. Four ejaculates were pooled and were split into four equal treatment aliquots. One of them was control (without CLC treatment, Group - I), other two aliquots were treated with 1 mg and 2 mg CLC/120 million buck spermatozoa (Group - II and Group - III) and last aliquot was treated with 1 mg MβCD/120 million spermatozoa (Group - IV), in TCG buffer and incubated for 15 min. After that each sample was diluted with TCG buffer containing 10% (v/v) egg yolk. Glycerolized diluents containing 5% (v/v) egg yolk and 12% (v/v) glycerol were added in three steps in 10 min interval 1 h after reaching 5°C to obtain 300 million progressive motile sperms/ml, packed into 0.5 ml French straws and kept for equilibration at 5°C for 2 h. The quality of equilibrated pre-freeze semen was analyzed by thawing one straw from each group was thawed in a water bath at 37°C for 30 seconds and assessing progressive motility, viability, acrosome integrity, and functional membrane integrity of spermatozoa.

The straws were frozen in liquid nitrogen vapor, 4 cm above the liquid nitrogen level, for 10 min and plunged into liquid nitrogen for storage. One straw from each group was thawed, after at least 24 h of freezing, in water bath at 37°C for 30 s and post-thaw semen quality (progressive motility, viability, acrosome integrity, and functional membrane integrity of spermatozoa in each group).

### Evaluation of spermatozoa

Percentage of progressive motile sperms were estimated by taking a 20 µl drop of diluted semen on a clean slide and examined under a microscope (×400). A minimum three fields were taken into account for determination of the percentage of progressive motile sperms. Functional membrane intactness of sperm was assessed by hypo-osmotic swelling (HOS) test described by Jeyendran *et al*. [11]. Two Eppendorff tubes, one containing 0.9 ml of HOS solution (100 mOsm/L), the other 0.9 ml of control solution (300 mOsm/L) were loaded with 0.1 ml of extended semen each and incubated at 37°C for 30 min. After incubation, a drop of eosin solution was added to the semen in Eppendorff tubes and a drop of the suspension from the bottom of each tube was taken on a clean, grease free glass slide and a smear were prepared. A minimum of 200 spermatozoa were examined under the high power objective (×400) of a microscope. The actual number of spermatozoa with intact plasma membrane was calculated by subtracting the number of positive spermatozoa in the control from HOS positive spermatozoa of the same semen sample.

To know the resistant of the sperm to incubation after thawing the semen sample of each group were incubated at 37°C up to 4 h of thawing and progressive motility and functional membrane integrity of spermatozoa in each group were assessed at 0 h, 30 min, 1 h, 2 h, 3 h, 4 h time interval as described below.

### Statistical analysis

Data were presented as means±standard error of mean. Percentage data (progressive motility, HOS positive sperm) was transformed using Arcsine prior to analysis, and the data were analyzed by one-way analysis of variance. Treatment were considered to be different at p<0.05.

## Results

During post-equilibration period spermatozoa treated with 1 mg and 2 mg CLC/120 million spermatozoa prior to cryopreservation showed significantly higher (p<0.05) percentage progressive motile sperms (Table-1) than spermatozoa of groups without CLC and MβCD containing group. Group treated with 1 mg of MβCD/120 million spermatozoa had significantly lower (p<0.05) percentage progressive motile sperms (Table-1) than other three groups. After cryopreservation, spermatozoa treated with 1 mg CLC/120 million spermatozoa before cryopreservation had significantly higher progressive motility when compared to the progressive motility of spermatozoa of other groups. Significantly lower percentage (p<0.05) motility of spermatozoa was observed among MβCD treated group when compared to other three groups after freezing and thawing (p<0.05).

**Table-1:** Effect of varying cholesterol levels in extender on pre-freeze and post-thaw progressive motility (per cent, Mean±SEM) of Malabari buck spermatozoa (n=12).

Stage of processing	Group I	Group II	Group III	Group IV
Pref-reeze	67.67±1.62^b^	75.17±0.68^a^	75.67±0.94^a^	53.5±3.78^c^
Post-thaw	40.50±1.65^b^	50.42±1.17^a^	44.58±2.11^b^	16.42±1.94^c^

Values having different superscript differ significantly (p<0.05). Group I: Control; Group II: Supplemented with CLC @ 1 mg/120×10^6^ spermatozoa; Group III: Supplemented with CLC @ 2 mg/120×10^6^ spermatozoa; Group IV: Supplemented with Methyl-β-cyclodextrin @ 1 mg/120×10^6^ spermatozoa

Functional membrane integrity of the spermatozoa treated with 0 mg and 2 mg CLC/120 million spermatozoa or 1 mg and 2 mg CLC/120 million spermatozoa before cryopreservation did not have significant difference during post equilibration period. Sperms treated with 1 mg MβCD/120 million spermatozoa prior to cryopreservation had significantly lower (p<0.05%) percentage of HOS positive spermatozoa than other three groups during post-equilibration and post-thaw stage (Table-2). Functional membrane integrity of the groups treated with 0 mg, 1 mg, 2 mg CLC/120 million spermatozoa did not differ significantly during post-thaw stage (Table-2).

**Table-2:** Effect of varying cholesterol levels in extender on pre-freeze and post-thaw HOS response (per cent, mean±SEM) of Malabari buck spermatozoa (n=12).

Stage of processing	Group I	Group II	Group III	Group IV
Pre-freeze	58.69±1.97^b^	65.58±1.68^a^	63.69±0.97^a,b^	46.63±2.48^c^
Post-thaw	43.61±0.92^a^	50.05±1.15^a^	49.14±1.19^a^	23.09±3.20^b^

Values having different superscript differ significantly (p<0.05). Group I: Control; Group II: Supplemented with CLC @ 1 mg/120×10^6^ spermatozoa; Group III: Supplemented with CLC @ 2 mg/120×10^6^ spermatozoa; Group IV: Supplemented with Methyl-β-cyclodextrin @ 1 mg/120×10^6^ spermatozoa

Spermatozoa treated with 1 mg CLC/120 × 10^6^ spermatozoa prior to cryopreservation maintained a significantly higher (p<0.05) percentage of progressive motile spermatozoa immediately after post-thawing and at 30 min of thawing than spermatozoa of other groups. CLC groups maintain a higher percentage of progressive motile spermatozoa up to 2 h of post-thawing. There was no significant difference between the spermatozoa of groups treated with CLC or without CLC at 3 and 4 h of incubation (Table-3).

**Table-3:** Effect of post-thaw incubation at 37°C on progressive motility (per cent, mean±SEM) of Malabari buck spermatozoa frozen in extenders with varying level of cholesterol (n=8).

Stage of incubation	Group I	Group II	Group III	Group IV
0 h	38.38±1.97^c^	52.5±2.11^a^	46.00±1.16^b^	13.69±1.39^d^
30 min	23.13±0.91^c^	41.00±1.48^a^	35.63±2.86^b^	5.00±0.82^d^
1 h	17.50±2.11^b^	28.88±1.48^a^	28.75±2.95^a^	3.38±0.82^c^
2 h	13.00±1.91^b^	23.38±2.98^a^	22.88±3.87^a^	1.38±0.73^c^
3 h	8.63±2.12^b^	13.75±2.61^a,b^	17.13±4.15^a^	0.38±0.37^b^
4 h	4.5±1.36^a,b^	10.75±2.65^a^	11.13±3.18^a^	0.00±0.00^b^

Values having different superscript differ significantly (p<0.05). Group I: Control; Group II: Supplemented with CLC @ 1 mg/120×10^6^ spermatozoa; Group III: Supplemented with CLC @ 2 mg/120×10^6^ spermatozoa; Group IV: Supplemented with Methyl-β-cyclodextrin @ 1 mg/120×10^6^ spermatozoa

## Discussion

Cryopreservation of spermatozoa is a great benefit to animal farming, agriculture and wildlife conservation strategy and simple alternative to embryo freezing for genetic conservation [12]. However, cryopreservation leads to extracellular and intracellular sperm damage result in lipid to protein reorganization and osmotic changes in the membrane.

Cholesterol is a predominant lipid of the sperm membrane and the cholesterol to phospholipid ratio is an important determinant of the sperm membrane fluidity [13]. Cholesterol modulates the fluidity of the membrane as temperature decreases during cryopreservation [14]. Treatment of spermatozoa with cholesterol has been found to reduce the sensitivity of sperm to cooling damage, membrane leakage and minimizing lateral phase separation of lipids [15]. Cyclodextrin, a cyclic oligosaccharide obtained by enzymatic degradation of starch, is used for incorporation or removal of cholesterol from cell membranes [16]. Treatment of spermatozoa with CLC have been shown to result in a 2-3 times increase in the sperm membrane cholesterol content than the normal spermatozoa and ultimately increasing cholesterol: phospholipid ratio [14].

The percentage of progressive motility of spermatozoa of the control group was significantly lower (p<0.05) than the progressive sperm motility observed among spermatozoa of the CLC treated groups. This is in accordance with the results obtained by Konyali *et al.*, in Murcino-Granadina buck and Yildiz *et al.*, in Carp sperm [8,17], Mocea *et al.*, in ram semen [18], Rebecca *et al.*, and Pamornsakda *et al.*, in stallion spermatozoa [19,20] and Crichton *et al.*, in dromedary camel spermatozoa [21] when treated with CLC before cryopreservation. The better post-thaw recovery of progressive sperm motility in spermatozoa treated 1 mg of CLC/120 million cells than control group without CLC is comparable to the values reported by Konyali *et al.*, [8] in bucks after treatment with 1 mg CLC/120 million sperm and Amidi *et al*. [22] after treatment with 1.5 mg CLC/120 million sperm and 2.5% bovine serum albumin. Better post-thaw progressive motility of buck spermatozoa, treated with CLC, was also observed by Farshad *et al.*, [23]. In addition to it a higher dose of CLC (2 mg CLC/120 million spermatozoa) put an inhibitory effect on spermatozoa ability for capacitation and to undergo acrosome reaction [24,25].

Purdy and Graham stated that CLC treated spermatozoa, which started with greater membrane cholesterol content prior to cooling, lost only a small amount of cholesterol during cryopreservation and had a greater amount of cholesterol after thawing than control sperm, which prevented premature capacitation and increased the longevity of cryopreserved sperms [14].

In the present study, treatment of spermatozoa with CLC was found to bring about an increased HOS response (Table-2) than the control group during pre-freeze stage. When sperms are diluted in a hypotonic solution, water moves into the cell fast causing increase in the cell volume which, may cause membrane damage and even cell lysis [26]. No significant difference was observed in the HOS response of spermatozoa in Group I, II, and III after freezing. Incorporation of cholesterol in the sperm membrane improves the sperm cryosurvival by increasing cholesterol: phospholipid ratio in the membrane which reduces lipid aggregation in specific domains within the membrane during phase transition from liquid to gel state and reduces the leakage of cellular components (such as potassium) from the cell [5]. Murphy *et al.*, 2014 and Ahmad *et al.*, 2013 demonstrated increased membrane fluidity obtained after adding CLC to stallion and ram respectively spermatozoa before cryopreservation [27,28].

Membrane damage occurs, when cell membrane undergoes a transition from liquid crystalline to gel [7]. Cold shock resulted in plasma membrane undergoing lipid phase transitions during the cooling process and was inversely correlated with the proportion of cholesterol within the plasma membrane [5]. Hartwig *et al.*, stated that when plasma membrane is exposed to low temperatures, the addition of sufficient levels of liposome containing cholesterol to sperm plasma membrane increases its cryoresistance and as a result it does not undergo a transition phase [29]. Thus, in the present study spermatozoa treated with CLC had better membrane cholesterol content and hence, could withstand cold shock better. Supplementation or removal of cholesterol in the extender for freezing did not manifest in any sperm abnormalities at pre-freeze stage.

Spermatozoa treated with MβCD showed significantly lower (p<0.05) percentage of progressive motile and intact plasma membrane than the other three groups during pre-freeze and post-thaw stage. Removal of cholesterol by MβCD has been shown to induce spermatozoa capacitation via tyrosine phosphorylation pathway, thereby shortening the sperm longevity [30]. Similar results were reported by Movassaghi *et al.*, in mouse spermatozoa [31]. The decreased recovery of post-thaw progressive motility in spermatozoa treated with MβCD is likely due to the removal of cholesterol from sperm membrane by cyclodextrin [32]. In addition, treatment with MβCD has been reported to induce sperm capacitation [30], prior to cooling, making the sperm more sensitive to damage.

CLC groups maintain a higher percentage of progressive motile spermatozoa up to 2 h of post-thawing. There was no significant difference between the spermatozoa of groups treated with CLC or without CLC at 3 and 4 h of incubation (Table-3). The better post-thaw recovery of progressive sperm motility in spermatozoa treated 1 mg of CLC/120 × 10^6^ cells than control group without CLC is comparable to the values reported by Konyali [8] in bucks after treatment with 1 mg CLC/120 × 10^6^ sperm and Amidi *et al.*, after treatment with 1.5 mg CLC/120 × 10^6^ sperm and 2.5% bovine serum albumin [22]. Better post-thaw progressive motility of buck spermatozoa, treated with CLC, was also observed by Farshad *et al*. [23]. Though cholesterol treatment was found to give better post-thaw motility during all stages of incubation when compared to the MβCD treated group; it was found that after 2 h of incubation, the spermatozoa treated with 1 mg of CLC/120 × 10^6^ spermatozoa had comparable motility with the control group, while the 2 mg CLC/120 × 10^6^ spermatozoa had better motility. By 4 h of incubation, even this motility was comparable to the control group motility. No references could be found supporting or explaining this observation. The possibility of metabolic products interfering with motility can be ruled out as the buffering capacity of the extended semen is expected to counter the products of metabolism. A possible explanation might be that spermatozoa subjected to cryopreservation have differing ability to withstand cryopreservation stress. In the control group, only the sturdy spermatozoa were able to survive the stress, while in the CLC treated group even the less sturdy spermatozoa survived the cryopreservation stress. However, these less sturdy spermatozoa would be subsequently losing their motility when incubated thus leading to a comparable motility after few hours of incubation.

## Conclusion

In conclusion, the percentage of progressive motile sperms was higher (p<0.05) in spermatozoa treated with 1 mg CLC/120 million sperms after cryopreservation. The spermatozoa treated with 1 mg CLC/120 million sperms was the optimal concentration for goat semen cryopreservation. Addition of MβCD might remove cholesterol from the sperm membrane making it more prone to cryodamage Future investigations may be directed at assessing the *in vivo* fertility of CLC treated spermatozoa in sexually healthy Malabari bucks.

## Authors’ Contributions

HMH and KNAG planned the study. BS carried out the work. HMH and KLB helped in carrying out the research. All authors participated in draft and revision of the manuscript. All authors read and approved the final manuscript.
